# Intimate Partner Violence Survivorship, Posttraumatic Stress Disorder and Disaster: Implications for Future Disasters

**DOI:** 10.1177/10778012231176205

**Published:** 2023-05-24

**Authors:** Clare E. B. Cannon, Regardt Ferreira, Fred Buttell, Allyson O’Connor

**Affiliations:** 1 Department of Human Ecology, 8789 University of California, Davis, CA, USA; 2 Department of Social Work, University of the Free State, Bloemfontein, South Africa; 3 School of Social Work, 5783 Tulane University, New Orleans, LA, USA; 4 Department of Social Work, Stellenbosch University, Stellenbosch, South Africa

**Keywords:** intimate partner violence, posttraumatic stress disorder, disasters, COVID-19, resilience

## Abstract

This study investigated posttraumatic stress disorder (PTSD) prevalence among a sample of intimate partner violence (IPV) survivors (*n*  =  77) who filed for restraining orders in rural Louisiana during the COVID-19 pandemic. IPV survivors were individually interviewed to assess their self-reported levels of perceived stress, resilience, potential PTSD, COVID-19-related experiences, and sociodemographic characteristics. Data were analyzed to differentiate group membership between two groups; non-PTSD and probable PTSD. Results suggest the probable PTSD group had lower levels of resilience and higher levels of perceived stress compared to the non-PTSD group. Findings suggest the importance of providing services during disaster to reduce PTSD for IPV survivors.

Research indicates that women with a history of intimate partner violence (IPV) experiences in the U.S. are more likely to experience depression, posttraumatic stress disorder (PTSD), and substance use disorder than those who have not suffered IPV (Dutton et al., 2006). Substantial extant research has found linkages between IPV and PTSD for multiple populations including women prisoners (Jones et al., 2021), women receiving treatment for IPV perpetration (Miles-McLean et al., 2021), women living in domestic violence shelters (Perez et al., 2012), women in college (Dardis et al., 2019), unemployed women (Kimerling et al., 2009), Latina survivors (Kelly, 2010), American Indian and Alaska Native survivors (Burrage et al., 2021), and African American women (Lilly & Graham-Bermann, 2009). The prevalence of PTSD among IPV victims varies widely from 33% to 80% (Jones et al., 2001). Research also suggests that experiences of IPV, including physical, sexual, and psychological forms of abuse, may vary by race and ethnicity (Shorey et al., 2021), and lead to the development of PTSD (e.g., Campbell, 2002; Dokkedahl et al., 2019; Dutton, 2009; Kelly, 2010; Nathanson et al., 2012; Perez et al., 2012; Woods et al., 2005). Though little research has focused on the relationships among race, PTSD, and IPV, Lilly and Graham-Bermann (2009) found that African American women reported lower levels of PTSD than their European American peers and this difference was observed despite the presence of more risk factors for African American women. Moreover, women who experience IPV often experience comorbid depression (Beydoun et al., 2012; Lilly & Graham-Bermann, 2009; Nathanson et al., 2012), and those who experience a greater severity of traumatic experiences tend to experience greater levels of depression (Campbell & Kendall-Tackett, 2005). IPV research further suggests that PTSD is key to the relationship between exposure to violence and adverse health outcomes (Dokkedahl et al., 2019; Dutton et al., 2006). For instance, Schnurr and Green (2004) have found that PTSD is a mediating pathway between violence and poor physical health.

At the same time, research suggests that resilience, understood as the ability to cope with adversity (Cutter, 2016), can play a role in reducing adverse outcomes from IPV (e.g., Hill et al., 2012; Howell et al., 2018; Jose & Novaco, 2016). Though the nature and definition of resilience continues to be debated given the ambiguity of the term, unclear differences in general or specific forms of resilience (Cavallo, 2014), disagreements over its interpretations (e.g., Cutter, 2016), and the perception of putting the responsibility of “bouncing back” on an individual rather than focusing on systemic structures (Moser et al., 2019), resilience continues to be an important concept particularly in regards to both exposure to disasters and personal violence. Much of the existing research suggests that individual resilience is associated with greater social support from friends and family and lower perceived stress (Jose & Novaco, 2016).

Though frequently studied in urban areas, IPV is also a problem for rural populations (Edwards, 2015; Peek-Asa et al., 2011; Sandberg, 2013; Shannon et al., 2006). While some research has found IPV occurs more frequently in rural locations compared to urban ones (Peek-Asa et al., 2011), other research suggests that IPV occurs at similar rates in rural and urban areas (Edwards, 2015). What seems to be clear is that the severity of IPV and worse psychosocial and physical health outcomes for rural residents may exist due to limited access to treatment and services (Edwards, 2015). At the same time, rural women are more likely than urban women to perceive the legal system as less helpful (Shannon et al., 2006). Yet, little research has focused on the relationship among PTSD and IPV for rural populations living in the U.S. (Cannon et al., 2023a).

Despite the empirical relationship between PTSD and IPV described above, little research has focused on the relationships among disasters, PTSD, and IPV (Bell & Folkerth, 2016; First et al., 2017; Gilmore et al., 2021; Harville et al., 2011; Lauve-Moon & Ferreira, 2017; Molyneaux et al., 2020; Resnick et al., 2020; Schumacher et al., 2010). Of the scant existing literature, Frasier et al. (2004) found no significant increase in IPV incidences following the flooding associated with Hurricane Floyd in 1999 in North Carolina. However, they did find that IPV victims consistently reported greater stress, PTSD symptoms, somatic and psychological difficulties and indicated that IPV victims may be at higher risk for stress-mediated chronic illnesses. Schumacher et al. (2010) did find that reports of IPV were associated with greater risk of PTSD and depression following Hurricane Katrina. Similarly, in their scoping review of women's mental health and IPV after disasters, Bell and Folkerth (2016) found support for the comorbidity of PTSD following disasters with increased prevalence for IPV survivors, particularly for women, who seem to be more vulnerable to both adverse outcomes from disasters and IPV than men. Research also suggests a history of IPV pre-disaster was associated with PTSD and depression symptoms with those having experienced IPV expressing more than three times the number of symptoms compared to those without IPV experiences (Resnick et al., 2020). While most of the disaster and IPV literature focuses on urban populations, there is some research into IPV, PTSD and disaster in rural areas. For instance, Molyneaux et al. (2020) in their study of violence and the 2009 bushfires in Victoria, Australia, found that IPV directed at women was significantly higher in areas with greater bushfire impacts, relative to areas with lower burn exposure.

Though research since the start of the COVID-19 pandemic has theorized and documented the increase in IPV due to stressors from the pandemic and public health measures, such as lock-downs, that trapped survivors with their abusers (e.g., Boserup et al., 2020; Buttell et al., 2021; Cannon et al., 2021; Ferreira et al., 2020; Kaukinen, 2020; Kofman & Garfin, 2020; Newnham et al., 2022; van Gelder et al., 2020), few studies have interviewed IPV survivors in rural areas to better understand their experiences during this infectious disease disaster (Cannon et al., 2023b). Women, as a social group in the U.S., are a particularly important population to study given the increased social, economic, health and psychological burdens they experienced due to the pandemic (Ayittey et al., 2020; Laster Pirtle & Wright, 2021; Preis et al., 2020; Raile et al., 2021), including, for instance, increased childcare demands given the widespread closure of daycares and schools for extended periods of time (Chotiner, 2020).

Taken together this literature indicates the need for further study into relationships among IPV, PTSD, resilience, and disasters particularly for rural populations, while recognizing such populations are racially diverse.

## Purpose of the Study

The aim of this study was to examine the prevalence of PTSD, perceived stress, and individual resilience during the COVID-19/Coronavirus Pandemic among a sample of IPV survivors who filed for a restraining order in a rural Louisiana parish (county) during the COVID-19 pandemic. Although research has debated the effectiveness of restraining orders by survivors of IPV (e.g., Benitez et al., 2010; McFarlane et al., 2004), restraining orders are often used by IPV survivors (Kanuha & Ross, 2004; Sorenson & Shen, 2005) as a tool to enhance their safety. This study seeks to better understand resilience, perceived stress, and COVID-19-related stressors for IPV survivors seeking legal protection.

## Method

This exploratory study involves a purposive sample of 77 participants who are survivors of IPV. Following the beginning of the lockdowns across the U.S. in March 2020, report logs from a local sheriff's victim assistance program were evaluated and IPV survivors were identified. Survivors were contacted and invited to participate in a structured interview conducted by phone from October 2020 to April 2022. As a vulnerable population, IPV survivors can be difficult to get in touch with and interview. This difficulty was compounded by the ongoing, unfolding COVID-19 pandemic, thus necessitating a long window of time to interview survivors. Structured interviews (e.g., Doody & Noonan, 2013; Ryan et al., 2009) were conducted and lasted between 30 and 60 minutes and were arranged at a safe and convenient time for survivors. The Tulane University Behavioral Institutional Review Board approved the study with the main inclusion criteria requiring that the survivors be 18 years of age or older, while also reporting a history of IPV. Exclusion criteria included participants who were younger than 18, who had not experienced IPV and had not field for a restraining order, and who did not live in one of the rural parishes supported by the sheriff's victim support program. The interview focused on capturing survivors’ (a) previous disaster experience, (b) resilience, perceived stress, and PTSD symptomology, (c) current situation as it relates to the COVID-19 pandemic, (d) experiences of IPV, and (e) personal demographics.

### Participants

The study sample consisted of individuals who experienced IPV, which resulted in their filing restraining orders from March 2020 to December 2021. The study sample was accessed through collaborations with a rural community-based Crisis Assistance Program located in a rural parish (county), in Southeast Louisiana that provides psychotherapy services to victims of criminal offenses. Survivors were accessed by a computer-generated list consisting of (i) the date the assault occurred, and the temporary restraining order was issued; and (ii) a contact phone number.

Each survivor from the logs was contacted by phone to participate in this study. Before initiating the interview tool, the interviewer (social worker) identified herself and informed the participant that the phone call had two purposes: (a) to assess their current situation and determine if immediate assistance was needed; and (b) to inquire if they were interested in participating in this study. The study sample includes 77 adults who completed the interview. SPSS 28 was utilized to conduct the final data analysis.

### Measures

#### Outcome variables

**Primary care PC-PTSD for DSM-5 (PC-PTSD-5).** This study used the Primary Care PTSD Screen for *DSM-5* (PC-PTSD-5). The scale consists of a five-item screen that was intended for use in PC settings (e.g., Schumacher et al., 2010). The scale starts with a screening item to assess if the respondent has had any exposure to traumatic events. In a case where a respondent answers with no exposure, the PC-PTSD-5 is complete and a score of 0 is assigned. In a case where a respondent indicates having experienced a traumatic event over the course of their life, the respondent is then asked to respond to a set of five additional yes/no questions about the trauma exposure and how the exposure has affected them over the past month. The cut-point of 3 on the PC-PTSD-5 is optimally sensitive to probable PTSD (Kar et al., 2021; Prins et al., 2016).

**Perceived Stress Scale.** This study also included the total score on the Perceived Stress Scale (PSS). The PSS has documented correlations with a variety of health-related measures including stress, self-reported health and health services, health behavior, smoking status and health seeking behaviors (Cohen, 1994). Scores on the PSS range from 0 to 40, with higher values on the PSS indicating greater perceived stress. Scores ranging from 0 to 13 are considered low, 14 to 26 moderate, and 27 to 40 as high. The PSS has been shown to have excellent psychometric properties across a wide range of research studies investigating stress (Lee, 2012; Roberti et al., 2006). Disaster research using the PSS has found ethnicity, previous disaster experiences, and mental health problems likely increased perceived stress (Schwartz et al., 2015).

**Individual resilience.** To gain a better understanding of the level of resilience amongst the study participants, this study employed the 10-item Connor Davidson Resilience Scale (CD-RISC 10). The CD-RISC 10 is known to be a well-established and abbreviated version of the original 25-item Connor Davidson Resilience Scale. The ordinal scale is a 5-point Likert scale ranging from 0 for “not at all” to 4 for “nearly all the time” (Connor & Davidson, 2003). The 10-item scale is widely acknowledged to have strong psychometric properties, across numerous demographic indicators, including gender, age, and race/ethnicity (Gulbrandsen, 2016; Liu et al., 2015; Ni et al., 2016; Windle et al., 2011). The scale is recognized for its construct validity, internal consistency and test-retest reliability (Connor & Davidson, 2003; Fincham et al., 2009; Nrugham et al., 2010). The scale asks respondents to rate their own resilience by responding to 10 statements, which can be found in Connor and Davidson (2003), such as : (1) “I am able to adapt when changes occur;” (2) “I can deal with whatever comes my way;” and (3) “I try to see the humorous side of things when I am faced with problems.” Scores on the CD-RISC 10 range from 0 to 40 with most studies using 32 as the cut-off score for being considered resilient.

#### Independent variables

**Demographic variables.** Leveraging prior research on social vulnerability to inform data collection during a disaster (e.g., Cannon et al., 2003), we included the following relevant demographic variables and response categories in the structured interview guide: (1) *age*; (2) *gender* (1  = * men*, 2  =  *women*, 3  =  *other*); (3) *sexual identity* (1  =  *heterosexual*, 2  =  *lesbian*); (4) *race* (1  =  *White*, 2  =  *Black/African American*, 3  =  *Mixed/Bi-Racial*, 4  =  *Native American*); (5) *relationship status* (1  =  *married*, 2  =  *widowed*, 3  =  *separated*, 4  =  *never been married but in a relationship*, 5  =  *divorced but in a relationship*, 6  =  *single*); (6) *employment* (1  =  *employed*, 2  =  *unemployed*, 3  =  *retired*, 4  =  *disability*); (7) *education* (1  =  *less than high school*, 2  =  *high school*, 3  =  *some college*, 4  =  *associate's degree*, 5  =  *bachelor's degree*, 6  =  *graduate degree*); (8) *English as a second language* (1 = *English*, 2  =  *English as second language*); (9) *residential status* (1  =  *own house*, 2  =  *rent*, 3  =  *other*); (10) *Rent/Mortgage Stress* (1  =  *stressed*, 2  =  *not stressed*); (11) *Nutritional Stress* (1  =  *stressed*, 2  =  *not stressed*).

**COVID-19 experiential variables.** The following set of variables and response categories explores experiences with the COVID-19 pandemic: (1) “Has COVID-19/Coronavirus lead to you losing your job?” (1  =  *yes*; 0  =  *no*); (2) “Has COVID-19/Coronavirus lead to you losing income?” (1  =  *yes*; 0  =  *no*); (3) “Do you think you will need help and cooperation from others (i.e., family, friends, or neighbors) to recover from the impact of COVID-19/Coronavirus”? (1  =  *very little help*, 2  =  *little help*, 3  =  *neither very little help nor very much help*, 4  =  *much help*, 5  =  *very much help*); (4) “Do you think you will need help and cooperation from others (i.e., government or non-governmental organizations) to recover from the impact of COVID-19/Coronavirus?” (1  =  *very little help*, 2  =  *little help*, 3  =  *neither very little help nor very much help*, 4  =  *much help*, 5  =  *very much help*); (5) “Have you experienced an increase in interpersonal conflict since COVID-19 started?” (1  =  *experienced an increase*, 2  =  *did not experience an increase*).

### Analytic Strategies

To investigate the study's research questions, we employed three different analytic techniques. First, we used Chi-squared test of association to determine whether there was an association between race (dichotomously coded as white and not white) and probable PTSD; second, we employed t-tests to determine whether there was a statistically significant relationship between (1) probable PTSD and individual resilience, and (2) probable PTSD and PSS. Finally, we conducted two binary logistic regressions in order to identify significant, explanatory variables related to stress exposure (i.e., PSS, rent/mortgage and nutrition stress) and experiences of the pandemic (i.e., the COVID-19 pandemic led to job loss or income loss, and there is anticipated need for family or governmental support to cope with these experiences) on probable PTSD group membership. Mathematically, the logistic binary regression estimates the probability of belonging to a group given a set of predictor or independent variables. This is defined as,

$$\pi (X)=\frac{\exp(\beta_{0}+\beta_{1}X_{1}+\ldots +\beta_{k}X_{k})}{1+\;\exp(\beta_{0}+\beta_{1}X_{1}+\ldots +\beta_{k}X_{k})}.$$

In this equation, π is the “success probability” or the likelihood that the observation is in the specified response category of the binary *Y* variable (Pardoe et al., 2022). Results of these analyses are reported below.

## Results

### Descriptive Statistics of the Sample

#### Descriptive statistics of socio-demographic characteristics

Table 1 provides a detailed description of the sample's demographic variables. The sample of 77 participants had a mean age of 39.05 (*SD*  =  13.08), with 92.2% females (*n*  =  71) and 5.2% males (*n*  =  4) with 1.3% identified as other (*n*  =  1) and 1.3% (*n*  =  1) trans: female to male, and 96.1% identified as heterosexual (*n*  =  74). Of the sample, 49.4% identified as white (*n*  =  38), 42.9% identified as Black or African American (*n*  =  33), 5.2% identified as bi-racial (*n*  =  4), 1.3% identified as Native American (*n*  =  1), and 1.3% identified as Other (*n*  =  1). In terms of relationship status, 45.5% (*n*  =  35) reported being in a relationship at the time of study participation. The majority of the sample reported being employed 55.8% (*n*  =  43) at the time of study participation. Regarding education, 10.4% of respondents had less than a high school diploma (*n*  =  8), 32.5% (*n*  =  25) reported having a high school education, 20.8% (*n*  =  16) reported some college, another 13% (*n*  =  10) reported holding an associate's degree, while 15.6% (*n*  =  12) held a bachelor's degree, and 7.8% (*n*  =  6) held a graduate degree. The majority of the sample spoke English at home (88.4%; *n*  =  68). Nearly two-thirds of the sample reported renting their home (62.3%; *n*  =  48). The majority of the sample expressed having worry or stress about being able to pay their rent or mortgage (70.2%; *n*  =  54). Of the sample, 46.6% (*n*  =  36) reported worrying that they did not have enough money to buy nutritious meals.

**Table 1.** Demographic Characteristics.

Characteristic	Participants (n =* * 77)		
	Mean/%	n/Range	*SD*
**Age (in years)**	39.05	41 19–76	13.08
Gender Male Female Other Trans—female to male	5.2 92.2 1.3 1.3	4 71 1 1	
Orientation Heterosexual Lesbian Bisexual	96.1 4.9 1.3	74 2 1	
Race White Black/African American Mixed/Bi-racial Native American Other	49.4 42.9 5.2 1.3 1.3	38 33 4 1 1	
Relationship Married Widowed Divorced Separated Never been married but in a relationship Divorced but in a relationship Single	15.6 5.2 5.2 16.9 20.8 9.1 27.3	12 4 4 13 16 7 21	
Employment Employed Unemployed Retired Disability	55.8 33.8 6.5 3.9	43 26 5 3	
Education Less than 12 years/no HS diploma HS Diploma/GED Some college Associate degree Bachelor degree Graduate degree	10.4 32.5 20.8 13.0 15.6 7.8	8 25 16 10 12 6	
English second language English English second language	88.4 11.6	68 9	
Residential Own house Rent/other	37.7 62.3	29 48	
Rent/mortgage stress Stressed/worried Not stressed/worried	70.2 29.8	54 23	
Nutritional stress Stressed/worried Not stressed/worried	46.7 53.3	36 41	
CD-RISC 10 mean score	27.7	77 9–40	7.4
Perceived Stress Scale	23.28	77 1–39	8.62
PC-PTSD-5 Probable PTSD No PTSD	57.1 42.9	44 33	

Regarding the resilience measure, the CD-RISC 10, respondents had a mean score of 27.7 (*SD*  =  7.4), below the oft-used cut-off score of 32 for resilience. According to the PSS, respondents had a mean score of 23.28 (*SD*  =  8.62), which falls on high end of the moderate range (i.e., 14–26). For the PC-PTSD-5, 57.1% (*n*  =  44) of respondents scored in the range of having probable PTSD.

#### COVID-19 pandemic experiential variables

This section provides an overview of experiences of the COVID-19 pandemic among the sample. The impact of the COVID-19 pandemic on the sample was extensive with 46.8% (*n*  =  36) reporting that the pandemic led to job loss. Of the sample, 64.9% (*n*  =  50) indicated they had lost income due to the pandemic and 36.4% (*n*  =  28) reported they will need social support, such as help and cooperation from others (e.g., family, friends, or neighbors), to recover from the impact of the pandemic. Regarding government and non-governmental support, 42.9% (*n*  =  33) of respondents indicated that they would need assistance from governmental entities to recover from the pandemic's impacts. COVID-19 experiences of the sample are reported in Table 2.

**Table 2.** COVID-19 Experiences.

Characteristic	Participants (n =* * 77)	
	Mean/%	n/Range
COVID-19 lead to losing job Lost job Did not lose Job	46.8 53.2	36 41
COVID-19 lead to losing income Losing income Did not lose income	64.9 35.1	50 27
Assistance from family/friends and neighbors Very little help Little help Neither very little help nor very much help Much help Very much help	44.2 16.9 2.6 11.7 24.7	34 13 2 9 19
Assistance from government & non-governmental organizations Very little help Little help Neither very little help nor very much help Much help Very much help	37.7 15.6 3.9 9.1 33.8	29 12 3 7 26

### Group Differences

#### Group differences for race and PTSD

Of the white survivors, 57.9% had probable PTSD, with 56.4% of racial minority survivors having probable PTSD. A Chi-squared test of association indicated that this difference was not significant (*χ*^2^_(1)_  =  .081, *p*  =  .482).

#### Group differences for probable PTSD and individual resilience

An independent sample *t*-test was performed to determine if there was a difference between survivors’ likelihood of having PTSD and those who do not have PTSD on the CD-RISC 10. The mean CD-RISC 10 score for the probable PTSD group was 25.68 (*SD*  =  7.53) and for the non-probable group, it was 30.39 (*SD*  =  6.39), indicating that the probable PTSD group had lower levels of resilience compared to the non-probable PTSD group. This difference was significant (*t*(75)  =  −2.962, *p*  =  .002) with a medium effect size (Cohen's *d*  =  −.666). Results are reported in Table 3.

**Table 3.** Group Differences for Probable PTSD and Individual Resilience.

CD-RISC 10	*n*	*M*	*SD*	*t*	*df*	*p*	Cohen's *d*
Probable PTSD**	44	25.68	7.53	−2.962	75	.002	−.666
Non-probable PTSD	33	30.39	6.39				

*Note*. *n * =  77.

*p < .05. **p < .01.

#### Group differences for probable PTSD and perceived stress

An independent sample *t*-test was performed to determine if there was a difference between survivors’ probable PTSD and those who did not have PTSD, on the PSS. The mean PSS score for the probable PTSD group was 26.13 (*SD*  =  7.18) and for the non-probable group was 19.48 (*SD*  =  9.0), indicating that the probable PTSD group had higher levels of stress compared to the non-probable PTSD group. This difference was significant (*t*(75)  =  3.605, *p*  =  .001) with a large effect size (Cohen's *d*  =  .830). Results are reported in Table 4.

**Table 4.** Group Differences for Probable PTSD and Perceived Stress.

Perceived Stress Scale	*n*	*M*	*SD*	*t*	*df*	*p*	Cohen's *d*
Probable PTSD***	44	26.13	7.18	3.605	75	.001	.830
Non-Probable PTSD	33	19.48	9.00				

*Note*. *n*  =  77.

*p < .05. **p < .01. ***p < .001.

### Logistic Regression Model: Does Stress Exposure Predict PTSD Group Membership?

A logistic regression analysis was performed to investigate how well perceived stress, measured by the PSS, rent/mortgage stress, and nutritional stress predict group membership based on probable PTSD (PC-PTSD-5). Collinearity statistics including variance inflation factor (VIF) and tolerance were conducted and no multicollinearity was found (analyses are available upon request). The model consisted of three predictors and allowed for simultaneous entry of all the independent variables. Using three predictor variables for this model satisfies the 10 cases per variable ratio suggested by Hair et al. (1998), all assumptions of logistic regression were met. The odds ratios of the logistic regression model are presented in Table 5.

**Table 5.** Logistic Regression Analysis of PTSD, Perceived Stress and Rent/Mortgage- and Nutrition Stress.

						95% CI for EXP (B)	
	B	SE	Wald	Exp (B)	Sig.	Lower	Upper
Perceived stress	.058	.038	2.306	1.059	.129	.983	1.141
Rent & mortgage stress*	−.523	.261	4.020	.593	.045	.356	.988
Nutrition stress	−.105	.266	.156	.900	.693	.535	1.516
Constant	.739	1.292	.327	2.094	.567		

*Note*. *n*  =  77. *df*  =  3.

*p < .05.

A test of the full model against a constant-only model was statistically significant (*x*^2^  =  20.865, df  =  3, *p*  =  .000). The model *R*² indicates that the model accounted for 32.2% of the total variance. Prediction success for the cases used in the development of the model had an overall success rate of 72.4%. Only one of the predictor variables, rent/mortgage stress (*Wald χ*^2^  =  4.020, df  =  1, *p*  =  .045, CI_.95_  =  .356, .988) was a statistically significant predictor of group membership based on probable PTSD. Based on the model, those respondents who reported rent/mortgage stress were 40% more likely to have reported probable PTSD compared to those in the non-probable PTSD group.

### Logistic Regression Model: Do COVID-19 Experiences Predict PTSD Group Membership?

A logistic regression analysis was performed to investigate how well COVID-19 experiences (i.e., “Has COVID-19 lead to you losing your job?” “Has COVID-19 lead to you losing income?” “How likely are you to need help and cooperation from others (e.g., family, friends, or neighbors) to recover from the impact of COVID-19?” and “How likely are you to need assistance from governmental entities to recover from the impact of COVID-19?”) predict group membership based on probable PTSD (PC-PTSD-5). The model consisted of four predictors and allowed for simultaneous entry of all the independent variables. Using four predictor variables for this model satisfies the 10 cases per variable ratio suggested by Hair et al. (1998), and all assumptions of logistic regression were met. A test of the full model against a constant-only model was not statistically significant (*x*^2^  =  7.210, df  =  4, *p*  =  .125).

## Discussion

The results of this study are important because they represent some of the first empirical evidence to date that gathers information about resilience, perceived stress, and potential PTSD from IPV survivors regarding their experiences with the COVID-19 pandemic. The data here suggest that the majority of participants reported probable PTSD. Roughly 57% of the sample scored in the range of likely having PTSD on the PC-PTSD-5. Given research that suggests IPV increases in prevalence and severity from exposure to disasters (Harville et al., 2011; Holmes et al., 2020; Schumacher et al., 2010), it is likely that the pandemic, as an infectious disease disaster, has increased IPV exposure, subsequently increasing PTSD and perceived stress (e.g., Bell & Folkerth, 2016; Schumacher et al., 2010), while decreasing individual resilience. We also find additional support for the differences in PTSD for IPV survivors with respect to race found in prior research (Shorey et al., 2021). Consistent with previous research that found African American women exhibited less PTSD than their white peers (e.g., Lilly & Graham-Bermann, 2009), our study found approximately 56% of racial minority IPV survivors interviewed expressed probable PTSD compared to 58% of white IPV survivors. It is important to note that though these findings are supported by the descriptive statistics of the study sample, the statistical test employed was not statistically significant (see the section “Limitations”).

This finding contributes to the chronically understudied factor of race in studying IPV survivors with implications for policy and interventions. Namely, IPV rates in the US suggest that African American women are at the highest risk for experiencing abuse from their intimate partners (U.S. Centers for Disease Control and Prevention, 2022). This might lead to an assumption that African American women experiencing a disaster, especially in a place like Louisiana with its history of racism and oppression, would be at greater risk for experiencing PTSD in a disaster. This assumption would prove faulty in this case and might lead to some misguided policy or programmatic solutions. For example, if it was assumed that African American women were at greater risk for PTSD post-disaster, outreach services and victim assistance programs would likely prioritize serving them initially, when what is needed is long-term support and redress of inequalities and oppression they may experience. Moreover, a strengths perspective, particularly one of posttraumatic growth (Anderson, 2018; Ferreira et al., 2018, 2019; First et al., 2018), might serve as a better explanatory model in this instance.

It seems likely that the African American women who comprised this sample had considerable experience navigating inequality and unfairness in Louisiana, as the income and demographic data for the state reveal stark racial disparities related to health (Louisiana Department of Health, 2021), environmental pollution (Terrell & James, 2022), and income inequality (Louisiana Budget Project, 2018), for example. In fact, prior research has found support for specific protective factors (i.e., greater spirituality, fewer violent partners, and greater social support) for African American women in bolstering their resilience to IPV (Howell et al., 2018). Prior research has found that though women survivors of IPV frequently report lower levels of perceived social support compared to non-abused women (Rodriguez et al., 2008), when survivors do experience social support it can bolster their resilience (Jose & Novaco, 2016; Oke, 2008), while social support can mitigate impacts to mental health (i.e., PTSD, depression) from experiences of IPV (e.g., Escribà-Agüir et al., 2010), and promote positive functioning in survivors (Ahmad et al., 2013). The findings, here, buttress the argument that the targets of helping efforts should be consulted in determining the priorities of what aid should be given and which problems should be addressed first (Cannon et al., 2023a). In this case, it seems that concerns stemming from the COVID-19 pandemic (e.g., stress exposure) might displace IPV concerns during a pandemic and its accompanying public health safety measures (e.g., lockdowns).

The results of the regression models have interesting implications for mitigating the impact of disasters. The first model suggests that pandemic related stressors are the biggest predictors of PTSD for this sample. In the model investigating various stressors, only rent/mortgage stress was a significant predictor of PTSD. Nonetheless, it was a robust model, correctly classifying 72% of the sample, explaining 32% of the variance, and indicating that respondents who reported rent/mortgage stress were 40% more likely to report probable PTSD. These results suggest that disaster policies related to an infectious disease disaster should target income maintenance and housing supports as a first priority to buffer against the probability of survivors developing PTSD. This proposition might have particular relevance for IPV survivors who frequently found themselves locked in and isolated with their abusive partners due to public health-related safety protocols (Kaukinen, 2020; Newnham et al., 2022).

Importantly, the current study adds to scant literature on disasters, PTSD, and IPV survivorship (Bell & Folkerth, 2016; Frasier et al., 2004; Molyneaux et al., 2020; Schumacher et al., 2010). These findings suggest that IPV survivors have to deal with a quadruple whammy—the disaster, increased stressors from the disaster, violence, and PTSD. These findings are particularly alarming in the context of the COVID-19 pandemic where the disaster has been ongoing for over 3 years. Public health safety protocols were isolating survivors at home, often times with their abusers (Lyons & Brewer, 2022), and reducing their access to resources, care, friends, and family who could potentially help reduce perceived stress, increase individual resilience, and somewhat prevent or alleviate PTSD (e.g., Barbara et al., 2020). Moreover, given research into the physical and mental health comorbid effects of PTSD and increased perceived stress, including depression and poor physical health (Beydoun et al., 2012; Schnurr & Green, 2004; Wright et al., 2019), IPV survivors are at greater risk of multiple and cascading adverse health outcomes from this dangerous confluence of factors.

### Limitations

This study focused on rural women who had experienced IPV and sought a protective order from the court. This research used a meaningful sampling strategy to investigate relationships among IPV, PTSD, and disasters in rural areas, however extrapolating findings to IPV survivors who do not file for restraining orders should be done so cautiously. This study used a cross-sectional design, which is important for gaining a better understanding of key dynamics among the relevant factors explored here but cannot determine causality. Though sufficient for the analytic techniques employed in this study, a larger sample could help further tease out relationships among PTSD and demographic and experiential variables, particularly differences with respect to race. These findings are important because they represent one of the first investigations into how rural women, in particular, who experienced IPV perceived the stressors related to the COVID-19 pandemic and how that might have translated into both PTSD risk and individual resilience. Given the differences described above, the relevance of these findings for urban women who experience IPV should be viewed cautiously.

## Conclusion

This study adds to the scant literature on rural women who experience IPV, while furthering our understanding of the complex relationship between disaster and IPV. This latter aspect is critically important, as very few studies have actually interviewed women about their IPV experiences during a pandemic. The findings here suggest that pandemic related concerns were strong predictors of the likelihood of developing PTSD, which has implications for approaches to future disasters and pandemics. A change in policies associated with the Violence Against Women Act and Victims of Crime Act to allow money to be deployed towards income maintenance and rent support during the next pandemic would likely be of immediate benefit to IPV survivors experiencing disaster.
